# Evaluation of ovarian reserve in women with thyroid autoimmunity

**DOI:** 10.5935/1518-0557.20240032

**Published:** 2024

**Authors:** Adriana Leal Griz Notaro, Filipe Tenório Lira Neto, Giuliano Marchetti Bedoschi, Maria Jéssica da Silva, Mariana Corrêa Nunes, Catharina Cavalcanti Pessoa Monteiro, José Natal Figueiroa, Alex Sandro Rolland Souza

**Affiliations:** 1 Instituto de Medicina Integral Prof. Fernando Figueira, Recife, PE, Brazil; 2 Amare Clinic, Recife, PE, Brazil; 3 Andros Recife Clinic, Recife, PE, Brazil; 4 Ribeirão Preto Medical School, University of São Paulo, Ribeirão Preto, SP, Brazil; 5 CM Medicina Reprodutiva, Recife, PE, Brazil

**Keywords:** autoimmune thyroiditis, Hashimoto’s disease, ovarian reserve, premature ovarian failure, fertility preservation

## Abstract

**Objective:**

To compare the ovarian reserve of women of reproductive age with and without thyroid autoimmunity (TAI).

**Methods:**

We performed a retrospective analysis of medical records from an assisted reproduction clinic from February 2017 to December 2021. Women aged between18 and 47 years with data on antithyroperoxidase and antithyroglobulin (anti-Tg) antibodies and assessment of ovarian reserve by anti-müllerian hormone (AMH) and antral follicle count (AFC) were included. Among the 188 participants included, 63 were diagnosed with TAI, and 125 had both antibodies negative. AMH and AFC were compared between groups. Subanalysis based on age, types of antibodies, and thyroid function markers were performed. In addition, bivariate analysis and regression models were used.

**Results:**

Overall, there was no difference in the median levels of AMH or AFC between the two groups. However, in the subgroup analysis by age, we observed a trend towards lower median levels of AMH in women over 39 years with TAI (0.9 ng/mL vs. 1.5 ng/mL, *p*=0.08). In a subanalysis according to antibodies, we found a significantly lower median AFC in the group with anti-Tg than in the group without this antibody (8.0 follicles vs. 11.5 follicles, *p*=0.036). We also found a significantly higher prevalence of anti-Tg in patients with low ovarian reserve compared to those with normal reserve (60.7% vs. 39.3%, *p*=0.038).

**Conclusions:**

The ovarian reserve of women with TAI appears to be insidiously compromised over the years, with a decreased ovarian reserve in women with anti-Tg.

## INTRODUCTION

Autoimmune thyroid diseases represent the most common autoimmune disorders in humans, especially in women of reproductive age (Orgiazzi, 2012; Jacobson *et al*., 1997; Golden *et al*., 2009). More often, this immune dysfunction selectively affects only the thyroid, but, in many cases, autoimmune thyroid diseases are associated with one or more organ-specific autoimmune dysfunctions. For instance, 12% to 40% of women with premature ovarian failure (POF) are diagnosed with autoimmune thyroid disease (Kirshenbaum & Orvieto, 2019).

The occurrence of POF is usually a gradual process characterized by a progressive decline in ovarian reserve. The mechanisms behind this decline are still being elucidated, but the most studied are a reduction in the number of primordial follicles, an accelerated process of follicular atresia and alterations in the recruitment and maturation of primordial follicles (Persani *et al*., 2010). The ovarian reserve represents the number of follicles still present in the ovaries. Characteristically, it is influenced by age, genetics, and environmental factors. The antral follicle count (AFC) and the anti-müllerian hormone (AMH) levels reflect the number of follicles still present in the ovaries and are the most frequently used tests to assess ovarian reserve (Tal & Seifer, 2017).

Although it is not yet possible to determine the rhythm of ovarian reserve decline on an individual level, knowledge of its behavior enables counseling on women’s reproductive planning. Gonadotoxic oncological treatments, genetic alterations such as mosaic for Turner Syndrome and Fragile X Syndrome, and benign pathologies, such as endometriosis and autoimmune oophoritis, constitute medical indications for fertility preservation, since they represent risk factors for a more accelerated impairment of follicle loss (Ferraretti *et al*., 2011). Therefore, in these cases, a more rigorous and specialized follow-up of these women should be indicated.

An association between thyroid autoimmune disease and premature ovarian failure has been reported by some authors (Dittmar & Kahaly, 2003; Sleiman *et al*., 2019; Dolmans & Manavella, 2019). There is evidence that antithyroid antibodies are found in the follicular fluid depending on their serum concentrations (Hoek *et al*., 1997). Although such intrafollicular antibodies may exert some influence on folliculogenesis, the mechanism of action in oocytes, granulosa cells, or ovarian stromal cells is still unclear (Betterle *et al*., 1993). In addition, the studies assessing the impact of TAI on the velocity of ovarian reserve decline have presented divergent results, and the current evidence does not enable a definitive conclusion regarding this association (Shah *et al*., 1995; Weghofer *et al*., 2016).

Therefore, it is of paramount importance to have a better understanding of the behavior of ovarian reserve in women with TAI in order to provide personalized reproductive planning and fertility preservation techniques before POF is established. This study aims to compare the ovarian reserve of women of reproductive age with and without TAI using AMH and AFC values.

## MATERIALS AND METHODS

### Study design and population

We retrospectively analyzed the medical records of patients who attended a referral clinic in assisted reproduction located in Recife, Brazil, during the period of February 2017 to December 2021.

### Participants

We included women between 18 and 47 years of age with data on the presence of antithyroid antibodies (routinely assessed at first consultation) and assessment of their ovarian reserve through AMH and AFC levels. Women with other possible causes of reduced ovarian reserve such as smoking, previous ovarian surgery, previous gonadotoxic treatment, previous pelvic or abdominal radiotherapy, chromosomal abnormalities, history of uterine artery embolization, diagnosis of non-autoimmune hypothyroidism, and Grave´s disease were excluded.

Participants positive for antithyroperoxidase (anti-TPO) and/or antithyroglobulin (anti-Tg) antibodies, according to the reference range of the laboratory where the test was performed, were considered to have TAI (Caturegli *et al*., 2014), while the control group was composed of women without these antibodies. Low ovarian reserve was defined based on the criteria adapted from POSEIDON (AMH < 1.2ng/mL and/or AFC < 5 follicles) (Humaidan *et al*., 2016). This classification enables the assisted reproduction specialist to estimate the number of eggs that can be obtained in an in vitro fertilization treatment, a data of great importance for the evaluation of the prognosis and reproductive counselling.

### Description of variables analyzed

The variables analyzed were age; body mass index (BMI), categorized according to the World Health Organization definition (WHO, 2000); reason for seeking the service; infertility; cause of infertility; endometriosis, diagnosed by ultrasound or magnetic resonance imaging; diagnosis of other autoimmune diseases; levels of thyroid-stimulating hormone (TSH); and free thyroxine (FT4).

Serum anti-TPO and anti-Tg levels were measured using electrochemiluminometric method and its results were recorded in UI/mL. Serum AMH levels were assessed at any stage of the menstrual cycle by the electrochemiluminometric method and recorded in ng/mL (Hehenkamp *et al*., 2006). The AFC was performed by the patient’s attending physician, a specialist in assisted reproduction, using transvaginal ultrasound during the initial follicular phase. All follicles measuring between 2 and 10 mm in both ovaries were counted (Tal & Seifer, 2017). When the AFC result was considered unexpected for the women’s age, the test was repeated in a subsequent cycle, and the highest count was considered for this study. The levels of TSH and FT4 were also measured by the electrochemiluminometric method and registered in mUI/L and ng/dL, respectively.

### Statistical analysis

The Statistical Package STATA 12.1SE (College Station, Texas, USA) was used for data analysis. The Kolmogorov-Smirnov test and quantile-quantile plot were combined to test for normality. Continuous variables with normal distribution were compared using the Student’s t-test and presented as means ± standard deviations. Continuous variables that were not normally distributed were compared with the Mann-Whitney U-test and presented as medians (interquartile ranges). Categorical variables were analyzed by the chi-square, Fisher’s exact test or Fisher-Freeman-Halton test, as appropriate and presented as counts (percentages). Spearman correlation coefficients were performed to test the association between numerical variables. Poisson multivariable regression models were performed to adjust for the effect of baseline characteristics. A value of *p*<0.05 was considered statistically significant.

### Ethical approval

This study was approved by the Research Ethics Committee of Instituto de Medicina Integral Prof. Fernando Figueira (CAAE 41181820.5.0000.5201; approval number: 4.486.991; date: 30/12/2020) and complied with the principles of the Declaration of Helsinki.

## RESULTS

**Figure 1:**
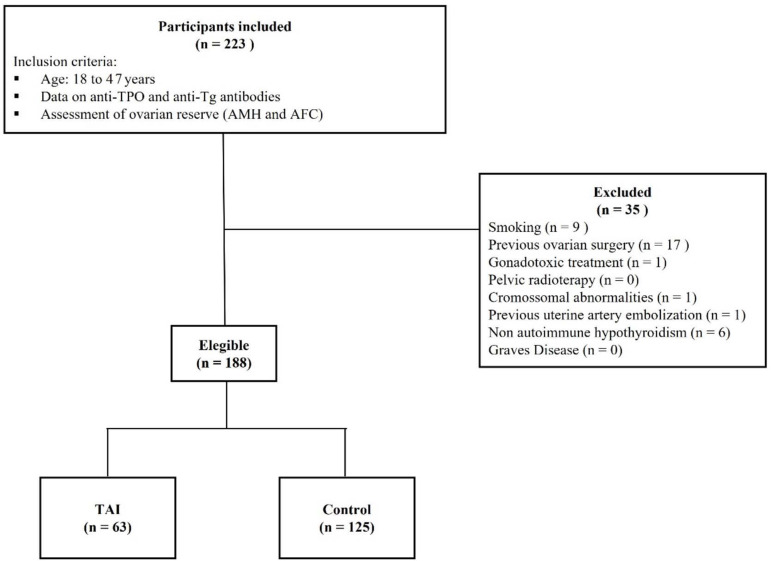
Participants capture flowchart. Anti-TPO=anti-thyroperoxidase; anti-Tg=anti-thyroglobulin; AMH=anti-Mullerian hormone; ACF=antral follicle count; TAI=thyroid autoimmunity.

A total of 188 women were eligible for analysis of their medical records, 63 (33.5%) of whom were diagnosed with TAI and 125 (66.5%) were controls (Figure 1). The mean age of the women included in the study was 36 years, with no difference between the groups. More than 50% of women were classified as having normal BMI, with no difference between women with TAI and the control group (Table 1).

**Table 1:** Baseline characteristics of participants.

Baseline characteristics	Overall	TAI	Control	*p* value
Participants (%)	188 (100)	63 (34)	125 (66)	NA
Age (years), mean ± SD	36.4±4.4	36.4±3.6	36.5±4.8	0.85^*^
BMI(Kg/m^2^) (%) < 18.5 18.5 - 24.9 25.0 - 29.9 ≥ 30	6 (3) 102 (57) 53 (30) 17 (10)	1 (2) 30 (53) 17 (30) 9 (16)	5 (4) 72 (60) 36 (30) 8 (7)	0.22^***^
Reason for seeking the clinic (%) Infertility Fertility preservation Recurrent miscarriage Reproductive evaluation Reproductive counseling Independent reproduction	135 (71.8) 24 (12.8) 7 (3.7) 10 (5.3) 11 (5.9) 1 (0.5)	45 (71.4) 9 (14.3) 3 (4.8) 2 (3.2) 4 (6.3) 0 (0.0)	90 (72) 15 (12.0) 4 (3.2) 8 (6.4) 7 (5.6) 1 (0.8)	0.914^**^
Infertility (%) Yes No	139 (73.9) 49 (26.1)	45 (71.4) 18 (28.6)	94 (75.2) 31 (24.8)	0.578^***^
Infertility cause (%) Ovulatory disfunction Tuboperitoneal factor/endometriosis Uterine factor Adenomyosis Male factor Unexplained infertility More than one associated factor	19 (13.7) 36 (25.9) 5 (3.6) 3 (2.2) 41 (29.5) 4 (2.9) 31 (22.2)	5 (26.3) 9 (25.0) 0 (0.0) 0 (0.0) 15 (36.6) 0 (0.0) 16 (51.6)	14 (73.7) 27 (75.0) 5 (100) 3 (100) 26 (63.4) 4 (100) 15 (48.4)	0.132^**^
Endometriosis (%) Yes No	48 (25.5) 140 (74.5)	14 (22.2) 49 (77.8)	34 (27.2) 91 (72.8)	0.460^***^
Other autoimmune disease (%) Yes No	7 (3.7) 181 (96.3)	2 (3.2) 61 (96.8)	5 (4.0) 120 (96.0)	> 0.99^****^

SD=Standard deviation; NA=not applicable.

* by Student’s t test;

** by Fisher-Freeman-Halton test;

*** by Chi-square test;

**** by Fisher´s exact test.

Regarding the reason for seeking the assisted reproduction clinic, in general, 71.8% of the women sought because of a diagnosis of infertility and there was no statistical difference between the groups. Likewise, no difference was found in the prevalence of the causes of infertility between the groups. In addition, the overall prevalence of endometriosis was 25.5%, and the diagnosis of other autoimmune diseases was 3.7%. Both variables had a similar distribution between the groups (Table 1). Furthermore, the median TSH levels, as well as the mean FT4 levels, were similar between the groups (Table 2).

**Table 2:** Thyroid function, ovarian reserve markers and antithyroid antibodies presence between TAI and control groups.

	TAI	Control	*p* value
TSH (mUI/L), median (IQR)	1.8 (1.1, 2.5)	1.8 (1.4, 2.5)	0.48^*^
Free T4 (ng/dL), mean ± SD	1.2±0.2	1.1±0.2	0.17^**^
AMH (ng/mL), median (IQR)	1.0 (0.6, 2.9)	1.5 (0.6, 3.1)	0.37^*^
AFC (number), median (IQR)	10 (7, 16)	11 (6, 19)	0.68^*^
Only anti-TPO positive (%)	56%	NA	NA
Only anti-Tg positive (%)	19%	NA	NA
Both anti-TPO and anti-Tg positive (%)	25%	NA	NA

TSH=thyroid-stimulating hormone; IQR=interquartile range; T4=thyroxine;

SD=standard deviation; AMH=anti-Müllerian hormone; AFC=antral follicle count; Anti-TPO=antithyroperoxidase;

Anti-Tg=antithyroglobulin; NA=not applicable.

* by Mann Whitney test;

** by Student’s t test;

*** by Fischer’s exact test.

Regarding the participants included in the TAI group, 56% presented only anti-TPO positive, 19% presented only anti-Tg positive and 25% presented both antibodies positive (Table 2). Concerning the ovarian reserve markers, no statistically significant differences were found when the medians of AMH (TAI: 1.0 ng/mL *vs*. control: 1.5 ng/mL, *p*=0.37) and AFC (TAI: 10 follicles *vs*. control: 11 follicles, *p*=0.68) were compared between groups (Table 2). Moreover, no correlations were found between ovarian reserve markers (AMH and AFC) and thyroid function (TSH and FT4) (Table 3). A multivariate regression analysis indicated age (*p*<0.001) and the presence of other autoimmune diseases (*p*=0.035) were the only variables independently associated with low ovarian reserve (Table 4).

**Table 3:** Spearman correlation coefficients between ovarian reserve markers and thyroid function markers.

	Overall (n=188)		TAI (n=63)		Control (n=125)	
	r_s_^*^	***p***	r_s_^*^	***p***	r^s *^	***p***
AMH vs. Free T4	-0.11	0.142	0.00	0.997	-0.16	0.082
AMH vs. TSH	0.02	0.750	0.10	0.450	-0.02	0.831
AFC vs. Free T4	-0.06	0.445	0.01	0.939	-0.09	0.311
AFC vs. TSH	0.11	0.396	-0.02	0.830	0.02	0.832

AMH=anti-Müllerian hormone; T4=thyroxine; TSH=thyroid-stimulating hormone; AFC=antral follicle count.

* *Spearman correlation coefficient*.

**Table 4:** Results of Poisson multivariable regression models to identify variables associated with ovarian reserve.

Variables	RR (95%CI)	p value
BMI (Kg/m^2^) Obesity No obesity	1.1 (0.71 - 1.73) 1.0	0.645
**Other autoimmune disease** Yes No	1.7 (1.04 - 2.70) 1.0	**0.035**
Endometriosis Yes No	1.1 (0.78 - 1.47) 1.0	0.671
Thyroid autoimmunity Yes No	1.2 (0.90 - 1.71) 1.0	0.183
**Age (years)** < 35 35 - 39 ≥ 40	1.0 2.07 (1.17 - 3.65) 3.8 (2.23 - 6.53)	**< 0.001**
TSH	1.12 (0.98 - 1.28)	0.108

RR=relative risk; CI=confidence interval; BMI=body mass index; TSH=thyroid-stimulating hormone.

When we performed a subgroup analysis by age, we observed that women aged over 39 years showed a trend of lower AMH levels in the TAI group when compared to women of the same age in the control group (0.9 ng/mL *vs*. 1.5 ng/mL, *p*=0.08) (Table 5). In addition, in the subanalysis based on the presence of antithyroid antibodies, a lower median AFC was found in the group that had the antithyroglobulin antibody in comparison to the participants without this antibody (8.0 follicles *vs*. 11.5 follicles, *p*=0.036) (Table 6). Similarly, when participants were classified according to ovarian reserve, those with low reserve had a higher prevalence of positive anti-Tg antibody compared to the group with normal ovarian reserve (60.7% *vs*. 39.3%, *p*=0.038) (Table 7).

**Table 5:** Ovarian reserve markers by age categories.

	TAI	Control	*p* value
**< 35 years (N)** AMH (ng/mL), median (IQR) AFC (number), median (IQR)	20 2.5 (1.2, 4.0) 15 (10, 24)	39 2.4 (1.3, 3.8) 18 (10, 21)	0.81^*^ 0.79^*^
**35 - 39 years (N)** AMH (ng/mL), median (IQR) AFC (number), median (IQR)	28 0.7 (0.2, 1.7) 10 (7, 15)	54 0.5 (0.3, 1.2) 11 (8, 21)	0.75^*^ 0.57^*^
**> 39 years (N)** AMH (ng/mL), median (IQR) AFC (number), median (IQR)	15 0.9 (0.6, 2.6) 6 (3, 8)	32 1.5 (0.8, 3.7) 5 (3, 10)	0.08^*^ 0.57^*^

AMH=anti-Müllerian hormone; IQR=interquartile range; AFC=antral follicle count.

* by Mann Whitney test

**Table 6:** Association between ovarian reserve markers and antithyroid antibodies.

Parameter	Anti-TPO +	Anti-TPO -	*p* value^***^	Anti-Tg +	Anti-Tg -	*p* value^***^
AMH^*^ median (IQR)	1.3 (0.6, 3.0)	1.5 (0.6, 3.1)	0.847	0.8 (0.6, 2.1)	1.5 (0.6, 3.1)	0.145
AFC^**^ median (IQR)	11.0 (7.0, 18.0)	10.0 (6.0, 19.0)	0.565	**8.0 (6.0, 11.0)**	**11.5 (7.0, 19.0)**	**0.036**

AMH=anti-Müllerian hormone; IQR=interquartile range; AFC=antral follicle count; Anti-TPO=antithyroperoxidase; Anti-Tg=antithyroglobulin.

* ng/mL;

** number;

*** Mann-Whitney’s test.

**Table 7:** Bivariate analysis of association between ovarian reserve and biological, clinical and laboratory variables.

Variable	Ovarian Reserve		RR (95%CI)	*p* value^**^
	Low^*^	Normal^*^		
TAI (%) Yes No	32 (50.8) 52 (41.6)	31 (49.2) 73 (58.4)	1.22 (0.89 - 1.68) 1.0	0.222
Endometriosis (%) Yes No	23 (47.9) 61 (43.6)	25 (52.1) 79 (56.4)	1.10 (0.77 - 1.56) 1.0	0.596
Other autoimmune disease (%) Yes No	5 (71.4) 79 (43.6)	2 (28.6) 102 (56.4)	1.64 (0.99 - 2.69) 1.0	0.053
Anti-TPO (%) Positive Negative	25 (49.0) 59 (43.1)	26 (51.0) 78 (56.9)	1.14 (0.81 - 1.60) 1.0	0.456
Anti-Tg (%) Positive Negative	17 (60.7) 67 (41.9)	11 (39.3) 93 (58.1)	1.45 (1.02 - 2.06) 1.0	0.038
Age (years) (%) < 35 35 - 39 > 39	12 (20.3) 36 (43.9) 36 (76.6)	47 (79.7) 46 (56.1) 11 (23.4)	1.0 2.16 (1.23 - 3.79) 3.77 (2.22 - 6.40)	<0.001 0.007 < 0.001
Obesity^***^ (%) Yes No	9 (52.9) 69 (43.8)	8 (47.1) 92 (57.1)	1.23 (0.76 - 2.0) 1.0	0.392

RR=relative risk; Anti-TPO=antithyroperoxidase; Anti-Tg=antithyroglobulin

* According to Poseidon´s criteria: low ovarian reserve=AMH < 1.2 ng/mL and/or AFC < 5 follicles;

** by Wald test;

*** Obesity=IMC ≥ 30 (Kg/m^2^)

## DISCUSSION

The present study compared the ovarian reserve of women of reproductive age with TAI (positive anti-TPO and/or positive anti-Tg) to the ovarian reserve of women who had negative antibodies. For this, we used the two main ovarian reserve markers, AMH and AFC; and we found an association between lower AFC and the presence of anti-Tg antibodies in women with TAI. In addition, when classifying women’s ovarian reserve as normal or low, we found a higher prevalence of positive anti-Tg in the low ovarian reserve group.

When a multivariate regression analysis was performed to reduce possible interference between the variables, we observed that age and the presence of other autoimmune diseases were independently associated with low ovarian reserve. However, as there were no differences regarding the mean age or the prevalence of other autoimmune diseases between the groups, this result does not compromise our analysis.

There are reports in the literature that 80% of women with idiopathic POF having a family or personal history of autoimmune diseases, and that 50% of them also have high levels of anti-Tg antibody (Košir Pogačnik *et al*., 2014). However, despite the association that may exist between POF and autoimmune thyroid disease, studies that assess the ovarian reserve of women with TAI have found contradictory results.

In our study, we observed that 19% of women with TAI were only positive for anti-Tg, a significant prevalence that reinforces the need for the evaluation of this antibody in the investigation of autoimmune thyroid disease. Other studies also evaluated the presence of both antibodies (Tuten *et al*., 2014; Saglam *et al*., 2015; Chen *et al*., 2017; Öztürk Ünsal *et al*., 2021; Korevaar *et al*., 2018; Ke *et al*., 2020; Osuka *et al*., 2018). However, among them, only two (Tuten *et al*., 2014; Ke *et al*., 2020) evaluated the two main markers of ovarian reserve for comparison between groups.

The study by Tuten *et al*. (2014) reported a positive correlation between AMH and antithyroid antibodies and no difference regarding AFC. A similar finding in women of reproductive age was published by Korevaar *et al*. (2018), who demonstrated higher AFC in women with TAI, but did not use the AMH as a marker of ovarian reserve. In the literature, only studies including adolescents described similar findings (Erol *et al*., 2016; Pirgon *et al*., 2016).

Conversely, the study by Ke *et al*. (2020) did not find an association between TAI and ovarian reserve markers; however, the mean age of women in this study was 30 years, which is lower than that in our study and in most studies that reported negative associations (Saglam *et al*., 2015; Chen *et al*., 2017; Bahri *et al*., 2019). Another important point in relation to this study is that the criteria used to classify low ovarian reserve was the FSH level, which is the latest marker to be affected, compared to AFC and AMH.

Another study included almost 5000 women and also did not find a link between TAI and low ovarian reserve (Polyzos *et al*., 2015). However, they did not investigate the anti-Tg antibody and evaluated AMH as a single marker of ovarian reserve. In addition, the study included mostly young women with a mean age of 32. A Japanese study also demonstrated no negative influence of the antibodies on the ovarian reserve; however, the authors reported a tendency towards lower levels of AMH in the presence of anti-TPO (Osuka *et al*., 2018). In contrast, several others found a negative association between antithyroid antibodies and ovarian reserve markers, as did our study (Saglam *et al*., 2015; Chen *et al*., 2017; Öztürk Ünsal *et al*., 2021; Bahri *et al*., 2019; Samsami *et al*., 2020).

Reviewing the literature, we found a high heterogeneity between studies regarding the diagnostic criteria for TAI (one or two antibodies) and the markers used to assess ovarian reserve. It is possible that this heterogeneity contributes to the diversity of results found. Another relevant factor, which apparently has contributed to the disagreement between our findings and those from some similar studies, is the age of the participants. A recent meta-analysis compared the AMH levels of women with TAI to control groups, stratifying the analyzes based on the age of the participants (Hasegawa *et al*., 2021). Five studies evaluated women of reproductive age and two included only adolescents. The results demonstrated that when only adult women were included, AMH levels tend to decline in the group with positive antibodies, whereas in the adolescent population, AMH levels were substantially higher in the group with positive antibodies. A limitation of this meta-analysis was the assessment of ovarian reserve only by AMH levels.

This limitation was addressed by a more recent meta-analysis that included 35 studies using AMH levels, AFC, and/or FSH levels to clarify the relationship between TAI and ovarian reserve (Li *et al*., 2022). The authors reported no significant difference between the ovarian reserve markers of women with TAI and the control group. However, in the subgroup analysis, women of reproductive age with TAI showed a statistically significant reduction in AMH levels and AFC, as well as higher baseline FSH levels when compared with the age-matched control group. In addition, there was an association between POF and the presence of anti-TPO in the group of women of reproductive age. Conversely, our study demonstrated a significantly higher prevalence of positive anti-Tg in the group with low ovarian reserve, with no difference in the prevalence of anti-TPO. However, the classification criteria used to characterize the group with a low ovarian reserve are not described in the meta-analysis, which could explain the difference in results.

The two aforementioned meta-analyses suggest that there is a negative correlation between TAI and ovarian reserve in adult women, concluding the opposite for the adolescent population, which seems to have a greater reserve. This reinforces the hypothesis that the impact of antibodies on ovarian reserve must be insidious, as shown by the trend towards lower levels of AMH in the group of women over 39 years of age found in our study. This data is important and should be further clarified in future studies, in view of the postponement of motherhood (Martin *et al*., 2012), giving these women the opportunity to evaluate fertility preservation options such as cryopreservation of eggs and embryos.

Our study evaluated the ovarian reserve of women with TAI through the two main markers of ovarian reserve (AMH and AFC); whereas most of the studies discussed above evaluated only one marker (Saglam *et al*., 2015; Chen *et al*., 2017; Öztürk Ünsal *et al*., 2021; Bahri *et al*., 2019; Polyzos *et al*., 2015; Osuka *et al*., 2018). Since there is evidence that a high percentage of women may present ovarian reserve markers with discordant results (Pastore *et al*., 2018), it is critical not to rely on just one of the makers in the clinical practice.

Another important strength of our study is that the diagnosis of TAI was performed by assessing the two main antithyroid antibodies (anti-TPO and anti-Tg), which makes the classification of women into the groups more reliable. Analyzing only one of the antibodies, as done in some studies (Bahri *et al*., 2019; Polyzos *et al*., 2015; Samsami *et al*., 2020), may lead to the inclusion of women with undiagnosed TAI in the control group, possibly compromising the results.

In our study, the median AMH levels found in the TAI group was 0.5 ng/mL lower than in the control group, a value that has clinical relevance, but was not statistically significant, perhaps due to our small sample size. Another limitation is the retrospective design of the study.

Regarding the variable “other autoimmune diseases”, although there is no specification about which disease it was, there was no difference in prevalence between the study group and the control group.

Ideally, longitudinal studies are needed to monitor the behavior of ovarian reserve markers over time in women newly diagnosed with TAI. This will clarify how ovarian reserves behave in these women and will help to develop a recommendation regarding reproductive planning for women diagnosed with autoimmune thyroid disease.

## CONCLUSION

Our study showed a trend towards lower AMH levels in women over 39 years of age with TAI compared to age-matched controls without TAI. In addition, we found lower AFC in women with anti-Tg, and that women with low ovarian reserve had a higher prevalence of this antibody. Thus, the ovarian reserve of women with TAI appears to be insidiously compromised over the years, with a decreased ovarian reserve in women with antithyroglobulin positive. To confirm our findings, longitudinal studies including women screened for the presence of both antibodies and with their ovarian reserve evaluated through more than one marker are needed.

From these findings, as the association of TAI with compromised ovarian reserve is plausible, maybe we can recommend that, once diagnosed with autoimmune thyroid disease, women of reproductive age should be referred for evaluation and reproductive counseling with an assisted reproduction specialist.

## References

[r1] Bahri S, Tehrani FR, Amouzgar A, Rahmati M, Tohidi M, Vasheghani M, Azizi F. (2019). Overtime trend of thyroid hormones and thyroid autoimmunity and
ovarian reserve: a longitudinal population study with a 12-year follow
up. BMC Endocr Disord.

[r2] Betterle C, Rossi A, Dalla Pria S, Artifoni A, Pedini B, Gavasso S, Caretto A. (1993). Premature ovarian failure: autoimmunity and natural
history. Clin Endocrinol.

[r3] Caturegli P, De Remigis A, Rose NR. (2014). Hashimoto thyroiditis: clinical and diagnostic
criteria. Autoimmun Rev.

[r4] Chen CW, Huang YL, Tzeng CR, Huang RL, Chen CH. (2017). Idiopathic Low Ovarian Reserve Is Associated with More Frequent
Positive Thyroid Peroxidase Antibodies. Thyroid.

[r5] Dittmar M, Kahaly GJ. (2003). Polyglandular autoimmune syndromes: immunogenetics and long-term
follow-up. J Clin Endocrinol Metab.

[r6] Dolmans MM, Manavella DD. (2019). Recent advances in fertility preservation. J Obstet Gynaecol Res.

[r7] Erol O, Parlak M, Ellidağ HY, Parlak AE, Derbent AU, Eren E, Yılmaz N. (2016). Serum anti-Müllerian hormone levels in euthyroid
adolescent girls with Hashimoto’s thyroiditis: relationship to antioxidant
status. Eur J Obstet Gynecol Reprod Biol.

[r8] Ferraretti AP, La Marca A, Fauser BC, Tarlatzis B, Nargund G, Gianaroli L, ESHRE working group on Poor Ovarian Response Definition (2011). ESHRE consensus on the definition of ‘poor response’ to ovarian
stimulation for in vitro fertilization: the Bologna criteria. Hum Reprod.

[r9] Golden SH, Robinson KA, Saldanha I, Anton B, Ladenson PW. (2009). Clinical review: Prevalence and incidence of endocrine and
metabolic disorders in the United States: a comprehensive
review. J Clin Endocrinol Metab.

[r10] Hasegawa Y, Kitahara Y, Osuka S, Tsukui Y, Kobayashi M, Iwase A. (2021). Effect of hypothyroidism and thyroid autoimmunity on the ovarian
reserve: A systematic review and meta-analysis. Reprod Med Biol.

[r11] Hehenkamp WJ, Looman CW, Themmen AP, de Jong FH, Te Velde ER, Broekmans FJ. (2006). Anti-Müllerian hormone levels in the spontaneous menstrual
cycle do not show substantial fluctuation. J Clin Endocrinol Metab.

[r12] Hoek A, Schoemaker J, Drexhage HA. (1997). Premature ovarian failure and ovarian
autoimmunity. Endocr Rev.

[r13] Humaidan P, Alviggi C, Fischer R, Esteves SC. (2016). The novel POSEIDON stratification of ‘Low prognosis patients in
Assisted Reproductive Technology’ and its proposed marker of successful
outcome. F1000Res.

[r14] Jacobson DL, Gange SJ, Rose NR, Graham NM. (1997). Epidemiology and estimated population burden of selected
autoimmune diseases in the United States. Clin Immunol Immunopathol.

[r15] Ke H, Hu J, Zhao L, Ding L, Jiao X, Qin Y. (2020). Impact of Thyroid Autoimmunity on Ovarian Reserve, Pregnancy
Outcomes, and Offspring Health in Euthyroid Women Following In Vitro
Fertilization/Intracytoplasmic Sperm Injection. Thyroid.

[r16] Kirshenbaum M, Orvieto R. (2019). Premature ovarian insufficiency (POI) and autoimmunity-an update
appraisal. J Assist Reprod Genet.

[r17] Korevaar TIM, Mínguez-Alarcón L, Messerlian C, de Poortere RA, Williams PL, Broeren MA, Hauser R, Souter IC. (2018). Association of Thyroid Function and Autoimmunity with Ovarian
Reserve in Women Seeking Infertility Care. Thyroid.

[r18] Košir Pogačnik R, Meden Vrtovec H, Vizjak A, Uršula Levičnik A, Slabe N, Ihan A. (2014). Possible role of autoimmunity in patients with premature ovarian
insufficiency. Int J Fertil Steril.

[r19] Li F, Lu H, Huang Y, Wang X, Zhang Q, Li X, Qiang L, Yang Q. (2022). A systematic review and meta-analysis of the association between
Hashimoto’s thyroiditis and ovarian reserve. Int Immunopharmacol.

[r20] Martin JA, Hamilton BE, Ventura SJ, Osterman MJ, Wilson EC, Mathews TJ. (2012). Births: final data for 2010. Natl Vital Stat Rep.

[r21] Orgiazzi J. (2012). Thyroid autoimmunity. Presse Med.

[r22] Osuka S, Iwase A, Goto M, Takikawa S, Nakamura T, Murase T, Kato N, Bayasula Kotani T, Kikkawa F. (2018). Thyroid Autoantibodies do not Impair the Ovarian Reserve in
Euthyroid Infertile Women: A Cross-Sectional Study. Horm Metab Res.

[r23] Öztürk Ünsal İ, Hepşen S, Akhanlı P, Çalapkulu M, Sencar ME, Yalçındağ A, Çakal E. (2021). Evaluation of serum anti-Müllerian hormone levels in women
with Hashimoto thyroiditis in the reproductive age. Turk J Med Sci.

[r24] Pastore LM, Christianson MS, Stelling J, Kearns WG, Segars JH. (2018). Reproductive ovarian testing and the alphabet soup of diagnoses:
DOR, POI, POF, POR, and FOR. J Assist Reprod Genet.

[r25] Persani L, Rossetti R, Cacciatore C. (2010). Genes involved in human premature ovarian failure. J Mol Endocrinol.

[r26] Pirgon O, Sivrice C, Demirtas H, Dundar B. (2016). Assessment of ovarian reserve in euthyroid adolescents with
Hashimoto thyroiditis. Gynecol Endocrinol.

[r27] NP Sakkas (2015). E, Vaiarelli A, Poppe K, Camus M, Tournaye H. Thyroid
autoimmunity, hypothyroidism and ovarian reserve: a cross-sectional study of
5000 women based on age-specific AMH values. Hum Reprod.

[r28] Saglam F, Onal ED, Ersoy R, Koca C, Ergin M, Erel O, Cakir B. (2015). Anti-Müllerian hormone as a marker of premature ovarian
aging in autoimmune thyroid disease. Gynecol Endocrinol.

[r29] Samsami A, Ghasmpour L, Moradi Alamdarloo S, Davoodi S, Rahmati J, Karimian A, Tavasoli M. (2020). Women with Autoimmune Thyroiditis have Lower Reproductive Life
Span or Not? A Cross- Sectional Study. Int J Community Based Nurs Midwifery.

[r30] Shah A, Mithal A, Bhatia E, Godbole MM. (1995). Extraovarian endocrine abnormalities in north Indian women with
premature ovarian failure. Natl Med J India.

[r31] Sleiman Z, Karaman E, Terzic M, Terzic S, Falzone G, Garzon S. (2019). Fertility Preservation in Benign Gynecological Diseases: Current
Approaches and Future Perspectives. J Reprod Infertil.

[r32] Tal R, Seifer DB. (2017). Ovarian reserve testing: a user’s guide. Am J Obstet Gynecol.

[r33] Tuten A, Hatipoglu E, Oncul M, Imamoglu M, Acikgoz AS, Yilmaz N, Ozcil MD, Kaya B, Misirlioglu AM, Sahmay S. (2014). Evaluation of ovarian reserve in Hashimoto’s
thyroiditis. Gynecol Endocrinol.

[r34] Weghofer A, Barad DH, Darmon S, Kushnir VA, Gleicher N. (2016). What affects functional ovarian reserve, thyroid function or
thyroid autoimmunity?. Reprod Biol Endocrinol.

[r35] WHO Consultation on Obesity (2000). Obesity: preventing and managing the global epidemic: report of a WHO
consultation.

